# The cytoskeleton controls membrane protein movement

**DOI:** 10.1093/plphys/kiae481

**Published:** 2024-09-11

**Authors:** Nicola Trozzi, Alicja B Kunkowska

**Affiliations:** Assistant Features Editor, Plant Physiology, American Society of Plant Biologists; Department of Computational and Systems Biology, John Innes Centre, Norwich Research Park, Norwich, NR4 7UH, UK; Department of Plant Molecular Biology, University of Lausanne, CH-1015 Lausanne, Switzerland; Assistant Features Editor, Plant Physiology, American Society of Plant Biologists; PlantLab, Institute of Plant Sciences, Sant’Anna School of Advanced Studies, 56010 Pisa, Italy

The plasma membrane acts as a dynamic interface between cells and their environment, hosting a diverse array of proteins crucial for cellular functions, including nutrient uptake, signal transduction, and responses to environmental stimuli (McFarlane et al. 2014; Wang et al. 2018). Their dynamics—including lateral mobility, clustering, and endocytosis—are key to plant adaptation and survival. While the fluid mosaic model once suggested free diffusion of these proteins, research has revealed a more nuanced picture of constrained mobility (Singer and Nicolson 1972; Kusumi et al. 2005). In plant cells, the cytoskeleton—composed of microtubules and actin filaments—influences cell shape, growth, and intracellular transport (Szymanski and Cosgrove 2009). However, its role in regulating plasma membrane protein dynamics has remained less clear (Lv et al. 2017; Luo et al. 2024).

In this issue of Plant Physiology, Luo et al. (2024) investigate how the cytoskeleton influences the behavior of plasma membrane proteins in *Arabidopsis thaliana*. The researchers examined 6 plasma membrane proteins representing different structural types: single transmembrane, multiple transmembrane, membrane-anchored, and glycosylphosphatidylinositol-anchored proteins (GPI-APs). Using structured illumination microscopy and variable-angle total internal reflection fluorescence microscopy, they observed distinct patterns of distribution and mobility for each protein type. Structured illumination microscopy provides high-resolution imaging beyond the diffraction limit. Variable-angle total internal reflection fluorescence microscopy allows visualization of molecules near the cell surface. To examine the cytoskeleton's influence, Luo et al. disrupted either microtubules or actin filaments using oryzalin and latrunculin B, respectively (Fig.).

**Figure.**
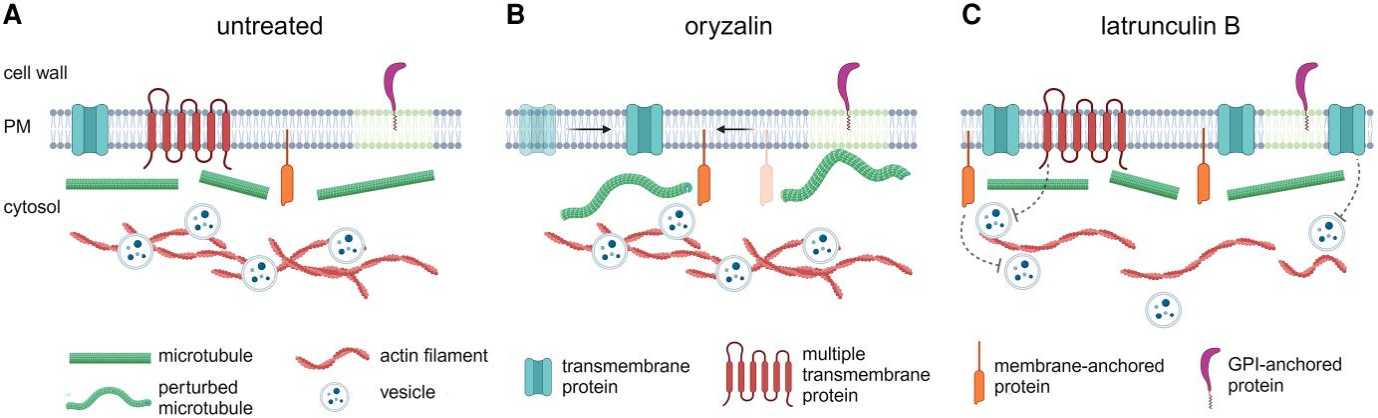
Schematic representation of the effects of oryzalin and latrunculin B on the movement of plasma membrane proteins. **A)** In untreated cells, the cytoskeleton serves as a barrier to the diffusion of plasma membrane proteins and facilitates the recruitment of cargo to vesicles. **B)** Treatment with oryzalin disrupts microtubules, leading to increased lateral mobility of plasma membrane proteins, particularly transmembrane and membrane-anchored proteins. **C)** Disruption of actin filaments by latrunculin B affects the internalization and trafficking of proteins. This leads to accumulation of most proteins at the plasma membrane, with the exception of GPI-anchored proteins. Created with BioRender.com.

Disruption of microtubules significantly increased the lateral mobility of most membrane proteins, with transmembrane and membrane-anchored proteins showing the most pronounced effects. The finding suggests microtubules act as diffusion barriers, corralling proteins into specific membrane domains. Actin disruption had a lesser impact on protein mobility but profoundly affected endocytic trafficking. By tracking the formation of brefeldin A bodies—structures that accumulate internalized proteins—the authors demonstrated that actin filaments are crucial for efficient clustering and internalization of membrane proteins. Disruption of actin filaments led to an accumulation of most proteins at the plasma membrane due to impaired internalization, effectively altering the protein composition of the cell surface. Intriguingly, GPI-APs showed minimal changes in mobility or endocytosis upon cytoskeleton disruption, suggesting a distinct regulatory mechanism possibly involving specialized membrane microdomains. The authors examined the endocytic process by analyzing colocalization of membrane proteins with clathrin light chain, a key component of clathrin-coated pits involved in endocytosis. Cytoskeletal disruption, particularly of actin filaments, reduced this colocalization, suggesting that the cytoskeleton facilitates the recruitment of cargo proteins to endocytic sites.

The findings on microtubule and actin disruption reveal the cytoskeleton as a key regulator of membrane protein dynamics. Microtubules appear to act as fences, compartmentalizing the membrane and restricting protein diffusion. This compartmentalization could facilitate protein–protein interactions, maintain polarized distributions, or regulate signaling pathways. By controlling the spatial organization and mobility of membrane proteins, the cytoskeleton may fine-tune signal transduction pathways, influencing how plants perceive and respond to their environment. For instance, the restricted diffusion of receptor proteins could affect their interaction with ligands or downstream signaling components, potentially modulating the sensitivity and specificity of cellular responses. Actin filaments, while less involved in constraining diffusion, play a critical role in organizing endocytosis. This is evidenced by the impaired clustering and internalization of membrane proteins observed after actin disruption. Actin may function by guiding proteins to internalization sites or facilitating vesicle formation, though the exact mechanisms require further investigation.

The study by Luo et al. opens several exciting avenues for research. One key question is how plants modulate cytoskeleton-membrane interactions in response to environmental stimuli. Do stress conditions alter these dynamics to reshape the membrane proteome? Another exciting area is the unique behavior of GPI-APs. What mechanisms control their mobility and endocytosis, and how do these unique behaviors relate to their functions? From a broader perspective, the Luo et al. (2024) paper underscores the importance of the cytoskeleton in cellular organization and function. By regulating membrane protein dynamics, the cytoskeleton influences processes ranging from nutrient uptake to signal transduction. This finding has potential implications for agricultural biotechnology. Could manipulating cytoskeleton–membrane interactions enhance crop stress tolerance or nutrient use efficiency?

The techniques employed in this study—particularly the combination of high-resolution imaging and targeted cytoskeletal disruption—provide a powerful toolkit for future investigations. These methods could be applied to study membrane dynamics in various plant tissues or developmental stages, offering a more comprehensive view of how cells organize their surfaces. In conclusion, the work by Luo et al. reveals the plant cytoskeleton as a crucial organizer, controlling the movements of membrane proteins. By constraining diffusion and facilitating endocytosis, the cytoskeleton helps coordinate the complex work of cellular communication and response.

## Data Availability

No data were generated or analyzed in this study.
